# Selective Audiovisual Semantic Integration Enabled by Feature-Selective Attention

**DOI:** 10.1038/srep18914

**Published:** 2016-01-13

**Authors:** Yuanqing Li, Jinyi Long, Biao Huang, Tianyou Yu, Wei Wu, Peijun Li, Fang Fang, Pei Sun

**Affiliations:** 1 Center for Brain Computer Interfaces and Brain Information Processing, South China University of Technology, Guangzhou, 510640, China; 2 Department of Radiology, Guangdong General Hospital, Guangzhou, 510080, China; 3 Department of Psychology and Key Laboratory of Machine Perception (Ministry of Education), Peking University, Beijing 100871, China; 4 Department of Psychology, School of Social Sciences, Tsinghua University, Beijing, 100084, China; 5 Guangzhou Key Laboratory of Brain Computer Interaction and Applications, Guangzhou 510640, China

## Abstract

An audiovisual object may contain multiple semantic features, such as the gender and emotional features of the speaker. Feature-selective attention and audiovisual semantic integration are two brain functions involved in the recognition of audiovisual objects. Humans often selectively attend to one or several features while ignoring the other features of an audiovisual object. Meanwhile, the human brain integrates semantic information from the visual and auditory modalities. However, how these two brain functions correlate with each other remains to be elucidated. In this functional magnetic resonance imaging (fMRI) study, we explored the neural mechanism by which feature-selective attention modulates audiovisual semantic integration. During the fMRI experiment, the subjects were presented with visual-only, auditory-only, or audiovisual dynamical facial stimuli and performed several feature-selective attention tasks. Our results revealed that a distribution of areas, including heteromodal areas and brain areas encoding attended features, may be involved in audiovisual semantic integration. Through feature-selective attention, the human brain may selectively integrate audiovisual semantic information from attended features by enhancing functional connectivity and thus regulating information flows from heteromodal areas to brain areas encoding the attended features.

An audiovisual object in the real world may contain multiple semantic features, such as the gender and emotional features of a speaker's face and voice. During the recognition of an audiovisual object, the human brain integrates the semantic information from these features obtained by the visual and the auditory modalities, i.e., audiovisual semantic integration may occur in the brain. Audiovisual integration facilitates rapid, robust and automatic object perception and recognition^1^,^2^,^3^. Comparisons of visual-only and auditory-only stimuli have revealed that congruent audiovisual stimuli lead to stronger neural responses than either type of stimulus alone in the posterior superior temporal sulcus/middle temporal gyrus (pSTS/MTG)^1^,^4^,^5^,^6^. Humans often selectively attend to a feature while ignoring the other features of the audiovisual object, and the attended feature is believed to be selectively processed in the brain. Behaviorally, the selection of features typically results in improved perceptual performance regarding the attended feature. These behavioral benefits have been linked to stronger or more consistent neuronal activity for the attended feature than the unattended feature^7^,^8^,^9^. Although audiovisual integration may occur at different levels, such as low-level audiovisual sensory integration, in which the semantic information of the audiovisual stimuli is not involved, and high-level audiovisual semantic integration^10^, studies of audiovisual semantic integration have been limited compared with studies of audiovisual sensory integration. In particular, the mechanisms underlying the modulation of audiovisual semantic integration in the brain by feature-selective attention have not been elucidated.

Previous studies have mainly focused on crossmodal attention and explored how crossmodal attention modulates audiovisual sensory integration across various stages. For example, in a study of the interactions between multisensory integration and attention using non-semantic visual-only, auditory-only and audiovisual stimuli^11^, an analysis of event-related potentials (ERPs) indicated that the effects of multisensory integration require that the multisensory objects be fully attended. The audiovisual sensory integration of motion information involves a specific network in which the parietal and perhaps lateral frontal cortices appear to be optimally situated to mediate the integration and attentional selection of motion information across modalities^12^. In audiovisual face perception, crossmodal attention influences crossmodal binding during speech reading^13^,^14^. Thus, attention and audiovisual integration interact with each other in a sophisticated manner. However, feature-selective attention in audiovisual conditions and the relationship between feature-selective attention and high-level audiovisual semantic integration remain to be explored.

In a single (visual or auditory) modality, feature-selective attention may lead to selective processing of the attended features of an object in the brain^7^,^8^,^9^,^15^,^16^,^17^. Nobre *et al.*^8^ demonstrated that ERPs are modulated by feature-selective attention and that irrelevant features are inhibited during the early stages of perceptual analysis in humans. In monkeys, Mirabella *et al.*^17^ observed that neurons in visual area V4 exhibit selectivity to elemental object features. Based on these studies that have employed unimodal stimuli, here we explore whether and how a similar feature-selective attention mechanism in an audiovisual condition is involved in audiovisual semantic integration.

We conjectured that feature-selective attention may function as a prerequisite for audiovisual semantic integration of the corresponding features of audiovisual stimuli and that the brain may selectively integrate the audiovisual semantic information about attended features. To test this hypothesis, we conducted a functional magnetic resonance imaging (fMRI) experiment in which the subjects were presented with visual-only, auditory-only or congruent audiovisual dynamic facial stimuli and instructed to perform four feature-selective attention tasks (number, gender, emotion, and bi-feature). Specifically, for the number task, a sequence of numbers were presented simultaneously with the visual-only, auditory-only, or audiovisual facial stimuli; the subjects were asked to attend to the numbers while ignoring all features of the facial stimuli. For the gender/emotion task, the subjects were instructed to selectively attend to a single semantic feature (emotion or gender) of the visual-only, auditory-only, or audiovisual facial stimuli. For the bi-feature task, the subjects attended to both the emotion and gender features. By applying a multivariate pattern analysis (MVPA) method to the collected fMRI data, we directly assessed the encoded semantic information of the emotion and gender features of the stimuli and analyzed the functional connectivity between the brain areas encoding a semantic feature and the heteromodal brain areas associated with audiovisual integration, the pSTS/MTG and perirhinal cortex^18^,^19^,^20^,^21^. We thus observed the different effects of audiovisual semantic integration produced by the four feature-selective attention tasks and generated new insights into the neural modulations of feature-selective attention on audiovisual semantic integration.

## Materials and Methods

### Subjects

Nine healthy native male Chinese (aged 21 to 48 years, with normal or corrected-to-normal vision) participated in the study. All participants provided informed written consent prior to the experiment. The experimental protocol was approved by the Ethics Committee of Guangdong General Hospital, China. The methods were performed in accordance with the approved guidelines.

### Experimental stimuli and design

**Figure 1:**
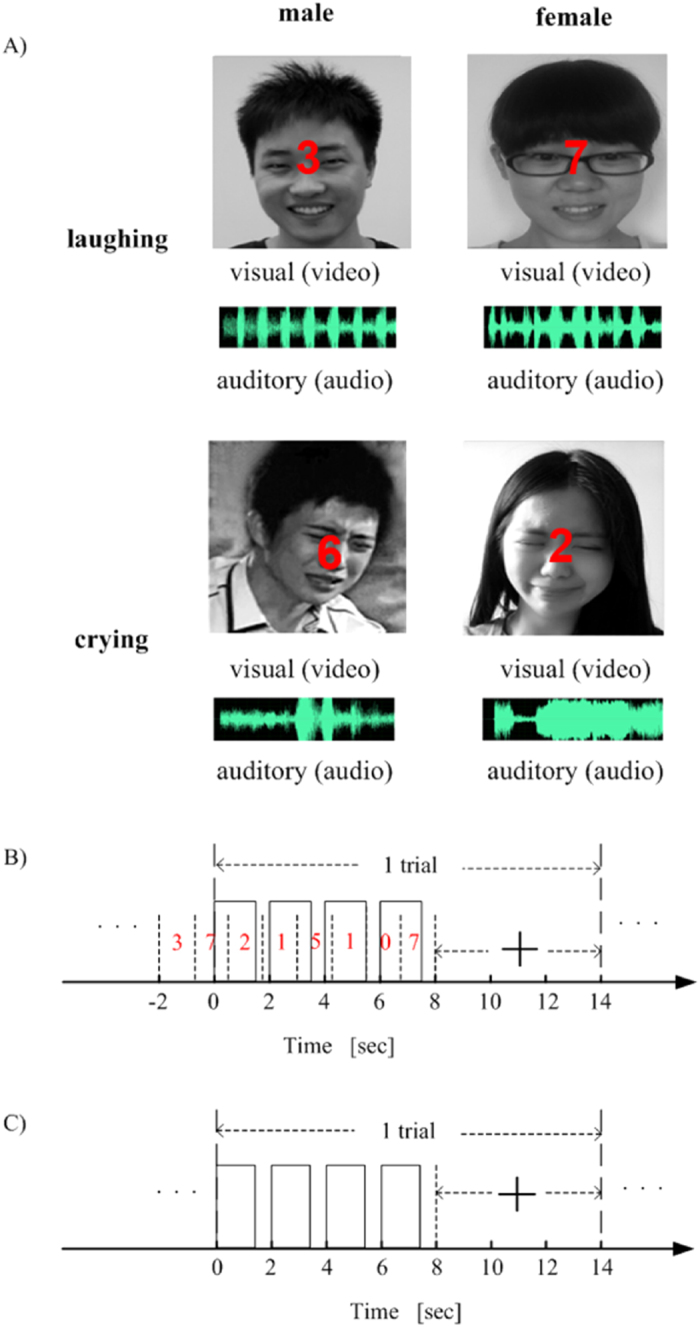
Experimental stimuli and time courses. (**A**) Four examples of audiovisual stimuli; the red numbers indicate runs with the number task only. (**B**) Time course of a trial for the runs with the number task, in which the stimuli included randomly presented numbers and videos/audios/movie clips. (**C**) Time course of a trial for the runs with the gender, emotion, or bi-feature task. For both (**B**,**C**), the presentation of a stimulus (video/audio/movie clip) lasted 1,400 ms and was repeated four times during the first eight seconds in a trial. A visual cue (“+”) appeared at the 8th second and persisted for six seconds.

The stimulus materials were the same as those in our previous study^22^. Specifically, we selected 80 movie clips that included video and audio recordings of Chinese faces from the Internet. These 80 movie clips, in which the faces were crying or laughing but did not speak, were semantically partitioned into two groups based on either gender (40 male vs. 40 female Chinese faces) or emotion (40 crying vs. 40 laughing faces). We further processed these stimulus materials using Windows Movie Maker. Each edited movie clip was in grayscale with a length of 1400 ms and was presented such that it subtended 10.7º × 8.7º. The luminance levels of the videos were matched by adjusting the total power value of each video (the sum of the squares of the pixel gray values; see examples in Fig. 1A without reference to the numbers). Similarly, the audio power levels were matched by adjusting the total power value of each audio clip. These edited movie clips consisting of both video and audio recordings were used as the audiovisual stimuli. Because the audiovisual stimuli were always congruent, we do not hereafter explicitly state the word “congruent” in the audiovisual stimulus condition. The unimodal visual/auditory stimuli were the videos/audios extracted from the above movie clips. During the experiment, an fMRI stimulation system (SA-9900 fMRI Stimulation System, Shenzhen Sinorad Medical Electronics Inc.) was used to present the visual and auditory stimuli in isolation and together. Specifically, the visual stimuli were projected onto a screen using an LCD projector, and the subjects viewed the visual stimuli through a mirror mounted on the head coil. The auditory stimuli were delivered through a pneumatic headset.

We utilized a 4×3 factorial experimental design with four experimental tasks associated with selective attention and three stimulus conditions for each task. Specifically, the four attentional tasks were as follows: the number task, attending to the numbers; the gender task, attending to the gender feature; the emotion task, attending to the emotion feature; and the bi-feature task, attending to both the gender and emotion features. The three stimulus conditions were as follows: visual-only (V), auditory-only (A), and audiovisual (AV). Table 1 shows the descriptions of the four attentional tasks, the corresponding experimental instructions, and the numbers of attended features of the visual-only, auditory-only, or audiovisual facial stimuli from the movie clips. Using different numbers of the attended features of the facial stimuli, we explored the effects of audiovisual integration on the neural representations of these features in this study. Furthermore, each subject performed 12 experimental runs corresponding to the 12 pairs of tasks and stimulus conditions, with the order pseudo-randomized (two runs per day). There were 80 trials in each run, corresponding to the 80 visual-only, auditory-only, or audiovisual stimuli. Each run included 10 blocks, and each block contained eight trials. At the beginning of the run, five volumes (corresponding to 10 seconds) were acquired without stimulation. The 80 stimuli (visual-only, auditory-only or audiovisual) were randomly assigned to the 80 trials in each run, with the gender and emotion categories of the stimuli balanced within each block. There was a 20-second blank period (gray screen and no auditory stimulation) between adjacent blocks. In the following, we first describe the experimental procedure for the runs with the number task.

For each of the three runs with the number task, in addition to the corresponding audiovisual, visual-only, or auditory-only facial stimuli from the movie clips, numbers in red appeared sequentially at the center of the screen (see Fig. 1A). The subject’s task was to attend to the numbers instead of the other stimuli (see Table 1). We designed a difficult number task for the subjects in which they were asked to find and count the repeated numbers to ensure that they fully ignored the features of the visual-only, auditory-only, or audiovisual facial stimuli. Therefore, the subjects performed this task with low accuracy, as shown in Fig. S3. At the beginning of each block, there were four seconds before the first trial, and a short instruction in Chinese (see Table 1) was displayed on the screen in the first two seconds (the last two seconds were used to display numbers, as indicated below). At the beginning of each trial, a visual-only, auditory-only or audiovisual facial stimulus was presented to the subject for 1,400 ms, followed by a 600-ms blank period. This two-second cycle with the same stimulus was repeated four times, followed by a six-second blank period. Therefore, one trial lasted 14 seconds. In addition to the above stimuli, eight numbers in red appeared one by one at the center of the screen, each a random integer from 0 to 9. Each number lasted 900 ms, and the interval between two subsequent numbers was 350 ms. The first number appeared 2 seconds before the beginning of this trial. The subjects were asked to find and count the repeated numbers. After the stimulation, a fixation cross appeared on the screen. The subjects then responded by pressing the right-hand keys according to the instruction for this block (see Table 1). The fixation cross changed color at the 12th second, indicating that the next trial would begin shortly (see Fig. 1B). In total, a run lasted 1,350 seconds.

The procedure for the three runs with the gender/emotion task was similar to that for the runs with the number task, except that no numbers appeared on the screen and the subjects performed a gender/emotion judgment task (See Table 1). Specifically, the subjects were asked to focus their attention on either the gender or the emotion of the presented stimuli (visual-only, auditory-only, or audiovisual facial stimuli; see Fig. 1A without regard to the numbers) and make a corresponding judgment (male vs. female for the gender task or crying vs. laughing for the emotion task) to each stimulus. At the beginning of each block, a short instruction (see Table 1) was displayed for four seconds on the screen. The time course of each trial was similar to that in the runs with number task (see Fig. 1C). In each trial, the subject was asked to judge the gender/emotion category of the stimulus and press the right-hand keys according to the instruction for this block.

For the three runs with the bi-feature task, the subjects were asked to simultaneously attend to both gender and emotion features (see Table 1). The experimental procedure for each run was similar to that for the runs with the gender/emotion task with the following differences. At the beginning of each block, a short instruction corresponding to the bi-feature task (see Table 1) was displayed on the screen for four seconds. The subject was required to simultaneously perform the gender and emotion judgments and press keys with both the left and right hands according to the presented instruction. Before the experiment, each subject was trained on a desktop computer until familiarity with the use of the left- and right-hand keys was attained.

### fMRI data collection

The fMRI data were collected using a GE Signal Excite HD 3-Tesla MR scanner at Guangdong General Hospital, China. Prior to the functional scanning, a 3D anatomical T1-weighted scan (FOV, 280 mm; matrix, 256 × 256; 128 slices; slice thickness: 1.8 mm) was acquired for each subject per day. During the functional experiment, gradient-echo echo-planar (EPI) T2*-weighted images (25 slices with ascending non-interleaved order; TR = 2,000 ms, TE = 35 ms, flip angle = 70°; FOV: 280 mm, matrix: 64 × 64, slice thickness: 5.5 mm, no gap) were acquired and covered the whole brain.

### Data processing

As described below, the data processing included four parts: data preprocessing, univariate general linear model (GLM) analysis, and two MVPA procedures. One MVPA procedure was for the reproducibility ratio and decoding accuracy calculations, and the other was for informative voxel localization and the cross-reproducibility ratio and functional connectivity calculations.

### Data preprocessing for each subject

In each run, the first five volumes collected before magnetization equilibrium was reached were discarded from the analysis. Next, the fMRI data collected in each run were preprocessed as follows: head motion correction, slice timing correction, co-registration between the functional scans and the structural scan, normalization to an MNI standard brain, data-masking to exclude non-brain voxels, time series detrending, and normalization of the time series in each block to zero mean and unit variance. All preprocessing steps were performed using SPM8^23^ and custom functions in MATLAB 7.4 (MathWorks, Natick, Massachusetts, USA).

### Univariate GLM analysis

This experiment included four experimental tasks (number, gender, emotion, and bi-feature). For each experimental task, three runs corresponding to the visual-only, the auditory-only, and the audiovisual stimulus conditions were performed. To confirm that audiovisual sensory integration occurred for each experimental task and determine the heteromodal areas associated with audiovisual integration, we performed voxel-wise group analysis of the fMRI data based on a mixed-effect two-level GLM in SPM8. In particular, using the data from the three number runs, we performed GLM analysis to explore the audiovisual integration at the sensory level when the subjects fully ignored the visual-only, auditory-only, or audiovisual facial stimuli while only attending to the numbers. The GLM analysis included the following data processing. The fMRI data for each subject were subjected to a first-level GLM, and the estimated beta coefficients across all subjects were then combined and analyzed using a second-level GLM. The following statistical criterion was used to determine brain areas for audiovisual sensory integration: [AV>max (A,V) (p < 0.05, FWE-corrected)] ∩ [V>0 or A>0 (p < 0.05, uncorrected)]^1^,^4^,^6^,^24^,^25^,^26^,^27^, where ∩ denotes the intersection of two sets. For each subject, each task, and each stimulus condition, we also computed the percent signal changes of the pSTS/MTG clusters via region-of-interest (ROI)-based analysis (implemented by the MATLAB toolbox MarsBaR-0.43^28^). Specifically, we identified the clusters consisting of significantly activated voxels in the bilateral pSTS/MTG via group GLM analysis as above. First, a GLM model was estimated from the mean BOLD signal of the clusters, and the percent signal change in the clusters was then computed as the ratio between the maximum of the estimated event response and the baseline.

### MVPA procedure for the calculation of the reproducibility ratio and decoding accuracy

For each subject, there were a total of 12 runs with four experimental tasks and three stimulus conditions. For each run, we calculated a reproducibility ratio corresponding to the gender feature and one corresponding to the emotion feature by applying an MVPA method to the fMRI data. The reproducibility ratio is an index that measures the similarity of the neural activity patterns within a class (e.g., the male class in the gender dimension) and the difference in neural activity patterns between two classes (e.g., male vs. female in the gender dimension). The higher the reproducibility ratio, the stronger the similarity of brain patterns within each class and the larger the difference between the two classes of brain patterns associated with the two gender or two emotion categories. Using the fMRI data, we also decoded the gender and emotion categories of the stimuli perceived by the subject. The neural representations of gender and emotion features were analyzed by comparing the reproducibility ratios or decoding accuracy rates for different stimulus conditions (visual-only, auditory-only, and audiovisual) and experimental tasks (number, gender, emotion, and bi-feature). In particular, the subjects only attended to the numbers during the three number runs, but the MVPA was based on the gender and emotion features of the visual-only, auditory-only, or audiovisual facial stimuli. In this manner, we analyzed the neural representations of gender and emotion features when none was attended. Below, we explain the MVPA procedure for gender categories (the MVPA procedure for emotion categories was similar).

For each run, 10-fold cross-validation was performed for the calculations of the reproducibility ratio and decoding accuracy corresponding to the two gender categories (refer to Fig. S1 in Supplemental Information). Specifically, the data from 80 trials were equally partitioned into 10 non-overlapping data sets. For the *k*th fold of the cross-validation (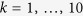), the *k*th data set (eight trials) was used for the test, and the other nine data sets (72 trials) were used for voxel selection and classifier training. After the 10-fold cross-validation, the average reproducibility ratio and decoding accuracy rate were calculated across all folds. The data processing procedure for the *k*th fold included the following:

#### 1) Voxel selection based on the training data

A spherical searchlight algorithm that was sequentially centered at each voxel with a 3-mm radius searchlight highlighting 19 voxels was applied to the training data set for voxel selection^29^. Within each searchlight corresponding to a voxel, we computed a Fisher ratio through Fisher linear discriminant analysis, and this ratio indicated the level of discrimination between the two gender categories in the local neighborhood of this voxel. A Fisher ratio map was thus obtained for the whole brain. *K* informative voxels with the highest Fisher ratios were then selected (e.g., *K* = 1500 in this study).

#### 2) Pattern extraction

Using the *K* selected voxels, we constructed a *K*-dimensional pattern vector for each trial of the training data in which each element represented the mean BOLD response of a selected voxel from the 6th to the 14th second of this trial (the last four volumes, to account for the delay in the hemodynamic response; each trial lasted 14 seconds). Similarly, we also extracted a *K*-dimensional pattern vector for each trial of the test data.

#### 3) Reproducibility ratio calculation for the test data

In this study, we used 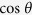 as a reproducibility index to assess the similarity of neural activity patterns elicited by the presented stimuli, where 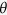 is the angle between two pattern vectors; the larger 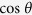, the higher the similarity. Specifically, for each trial of the test data of the *k*th fold of the cross-validation, we obtained a pattern vector denoted by a column vector 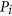 that belonged to either of the two classes denoted by 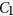 and 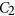 (male vs. female in the gender dimension). There were 8 pattern vectors corresponding to the 8 trials from the test data, of which 4 belonged to 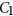 and the other 4 belonged to 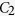. We calculated the average within-class and between-class reproducibility indices 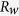 and 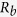 and the reproducibility ratio 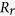 for the *k*th fold of cross-validation as follows:


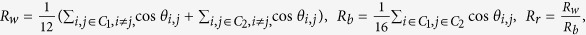
where 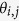 is the angle between two pattern vectors 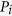 and 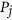. Note that there were 6 pairs of different patterns 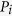 and 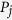 for each class of 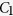 and 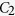. Thus, there were 12 pairs of different patterns 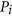 and 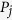 belonging to the same class (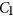 or 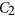). Furthermore, there were 16 pairs of different patterns 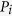 and 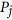 belonging to classes 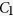 and 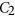
respectively.

#### 4) Classifier training based on the training data

For gender category prediction, a linear support vector machine (SVM) classifier was trained based on the pattern vectors with labels of the training data.

#### 5) Category prediction for the test data

The gender category of each trial of the test data was then predicted by applying the trained SVM to the corresponding pattern vector.

### MVPA procedure for informative voxel localization and the cross-reproducibility ratio and functional connectivity calculations

In this MVPA procedure, we first used the data from the audiovisual run with the bi-feature task to localize an informative voxel set separately for the two gender or two emotion categories. Based on this informative voxel set, we calculated the cross-reproducibility ratio and functional connectivity for each audiovisual run with number, gender and emotion tasks. The neural representations of the gender or emotion feature were further analyzed by comparing cross-reproducibility ratios and functional connectivity with different attentional tasks (refer to Fig. S2 in Supplemental Information). The data processing in this MVPA included the following:

#### 1) Localization of informative voxels

Using the labeled data from the audiovisual run with the bi-feature task, we obtained two voxel sets, denoted as Set 1 and Set 2, which were informative for the two gender and the two emotion categories, respectively. Here, the localization of Set 1 is described as an example. For each subject, we performed a 10-fold cross-validation for gender category decoding, as described above. Based on the SVM training in each fold, we obtained a SVM weight map (the unselected voxels were assigned a weight of zero). The SVM weights reflected the importance of voxels for decoding. By averaging the weight maps across all folds and all subjects, an actual group weight map was obtained for the gender category differentiation. Next, we performed 1,000 permutations to obtain 1,000 group weight maps for the gender categories. Each group weight map was constructed similarly to the above method except that for each subject, the labels of all the trials were randomly assigned. To control the family-wise error (FWE) rate, a null distribution was constructed using the 1,000 maximum voxel weights, each of which was from a group weight map^30^. Thresholding the actual group weight map using the 95th percentile of the null distribution, we obtained the informative voxel Set 1, corresponding to the gender categories.

#### 2) Cross-reproducibility ratios

For each of the three audiovisual runs with number, gender and emotion tasks, we separately calculated the reproducibility ratios for the gender categories and the emotion categories using the localized voxel Sets 1 and 2, using methods similar to those described above.

#### 3) Functional connectivity analysis

For each of the audiovisual runs with number, gender and emotion tasks, we first conducted a multivariate Granger causality analysis to calculate the functional connectivity between the brain areas related to gender category differentiation and the heteromodal areas^31^,^32^. Specifically, using the data from the audiovisual run with the bi-feature task, we obtained the informative voxel set, namely, Set 1, for the gender categories. Consistent with previous reports^10^,^32^, we identified four heteromodal areas: the left pSTS/MTG, the right pSTS/MTG, the left perirhinal cortex, and the right perirhinal cortex. In particular, The perirhinal cortex integrates the audiovisual information associated with semantic features into higher-level conceptual representations^10^. For each pair of clusters, one from Set 1 and the other from one of the four heteromodal areas, we obtained two average time series by averaging the time series of all of the voxels within each of the two clusters. We then calculated the functional connectivity by performing the Granger causal analysis at the significance level 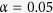 (FDR correction)^33^. For each of the audiovisual runs with the number, gender and emotion tasks, we also calculated the functional connectivity between the brain areas related to emotion category differentiation and the heteromodal areas using procedures similar to those described above.

## Results

The behavioral results from the fMRI experiment are presented in Fig. S3. In the following section, we present the results of our fMRI data analysis.

### Brain areas associated with audiovisual integration at the sensory level

**Figure 2:**
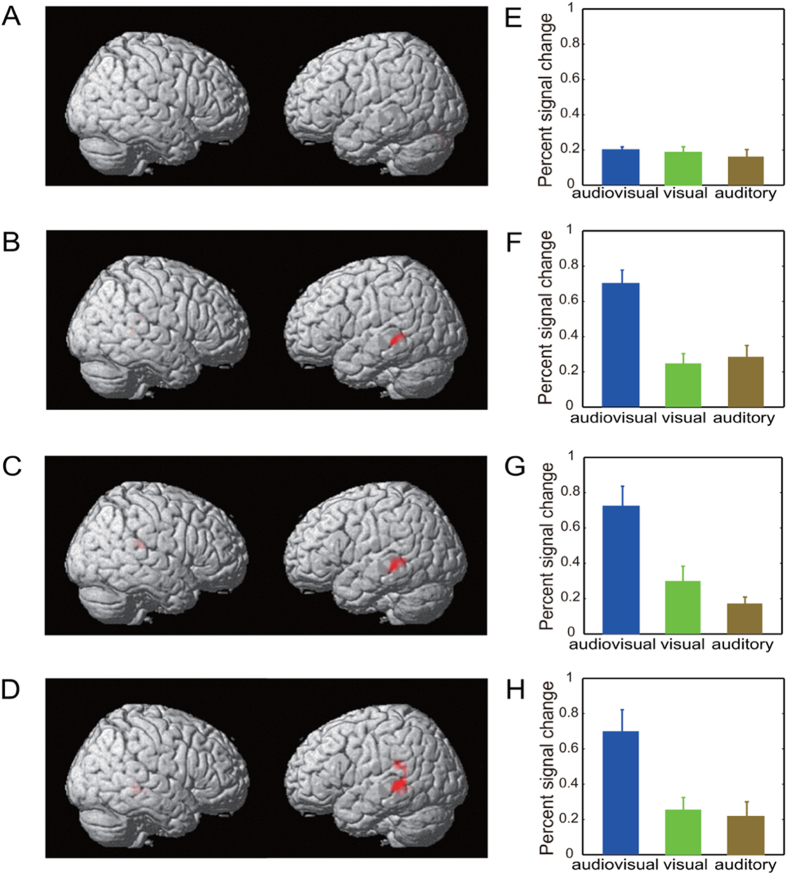
Brain areas for audiovisual sensory integration that met the criterion [AV>max (A,V) (p < 0.05, FWE-corrected)]∩[V>0 or A>0 (p < 0.05, uncorrected)]. (**A**) No brain areas exhibited audiovisual sensory integration for the number task. (**B**) Brain areas exhibiting audiovisual sensory integration for the gender task, including the left pSTS/MTG (Talairach coordinates of the cluster center: (−57, −34, −5); cluster size: 76). (**C**) Brain areas exhibiting audiovisual sensory integration for the emotion task, including the left pSTS/MTG (cluster center: (−60, −40, 1); cluster size: 98) and the right pSTS/MTG (cluster center: (45, −34, 19); cluster size: 13). (**D**) Brain areas exhibiting audiovisual sensory integration for the bi-feature task, including the left pSTS/MTG (cluster center: (−54, −46, 4); cluster size: 105) and the right pSTS/MTG (cluster center: (61, −45, −7); cluster size: 13). (**E**–**H**): Percent signal changes evoked by the audiovisual, visual-only and auditory-only stimuli in the bilateral pSTS/MTG activation clusters shown in (**A–D**), respectively (the percent signal changes in (**E**) were calculated using the union of all activated voxels shown in (**B**–**D**).

In this experiment, there were a total of four attentional tasks: number, gender, emotion, and bi-feature. For each experimental task, there were three runs corresponding to the visual-only, auditory-only, and audiovisual stimulus conditions (see Materials and Methods). To confirm that audiovisual sensory integration occurred for each experimental task, we performed GLM analysis of the fMRI data at the group level and identified the heteromodal areas pSTS/MTG exhibiting enhancement of neural responses in the audiovisual condition (see Materials and Methods). These results are presented in Fig. 2, which reveals that audiovisual sensory integration occurred for the gender, emotion, and bi-feature tasks but not for the number task. However, one-way repeated measures ANOVA indicated that there was no significant difference among the percent signal changes for the gender, emotion, and bi-feature tasks in the audiovisual stimulus condition (p = 0.96, F(2, 8) = 0.04, see subplots F, G, and H in Fig. 2). That is, based on the neural response levels in the pSTS/MTG, the effects of audiovisual integration cannot be differentiated for different experimental tasks or different semantic features. Thus, audiovisual sensory integration rather than audiovisual semantic integration occurred in the identified heteromodal areas of the pSTS/MTG, consistent with previous results^10^.

### MVPA results of the reproducibility ratios and decoding accuracy rates

**Figure 3:**
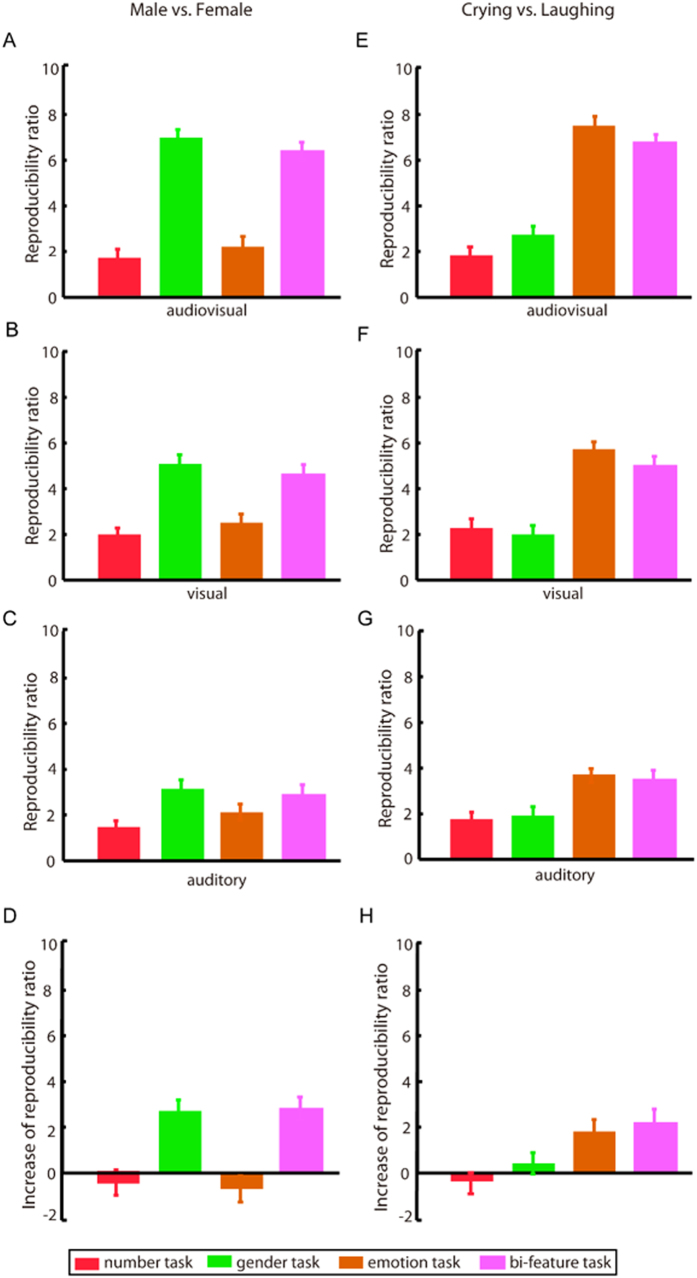
Reproducibility ratios (means and standard errors across all subjects) and the corresponding comparison results. Left/Right: gender/emotion categories; the first 3 rows: audiovisual, visual-only, and auditory-only stimulus conditions, respectively; the 4th row: the reproducibility ratio in the audiovisual condition minus the maximum of the reproducibility ratios in the visual-only and auditory-only conditions.

Using an MVPA method, for each of the 12 runs of the experiment with four attentional tasks and three stimulus conditions, we calculated two reproducibility ratios corresponding to the gender categories (“male” vs. “female”) and the emotion categories (“crying” vs. “laughing”) of the stimuli respectively. Furthermore, each calculation of reproducibility ratio was based on 1500 selected voxels (see Materials and Methods); the results of reproducibility ratios are shown in Fig. 3. We also systematically varied the number of selected voxels from 25 to 1500 to calculate the reproducibility ratios and obtained similar results (see Fig. S4).

For the reproducibility ratios of the gender/emotion categories, two-way repeated measures ANOVA revealed significant main effects of stimulus condition (gender categories: p < 10^−17^, F(2, 8) = 88.73; emotion categories: p < 10^−16^, F(2, 8) = 51.37) and experimental task (gender categories: p < 10^−17^, F(3, 8) = 81.13; emotion categories: p < 10^−17^, F(3, 8) = 68.26) (Fig. 3A–C,E–G). There was also a significant interaction effect between the two factors of stimulus condition and experimental task (gender categories: p < 10^−17^, F(6, 8) = 30.07; emotion categories: p < 10^−8^, F(6, 8) = 10.05). Post hoc Bonferroni-corrected paired t-tests on the stimulus conditions revealed the following: (i) for each task-relevant feature (gender categories with the gender or the bi-feature task, left panel of Fig. 3; emotion categories with the emotion or the bi-feature task, right panel of Fig. 3), the reproducibility ratios were significantly higher for the audiovisual stimulus condition than for the visual- or auditory-only stimulus condition (all p < 0.001 corrected); and (ii) for each task-irrelevant feature (gender categories with the number or the emotion task, left panel of Fig. 3; emotion categories with the number or the gender task, right panel of Fig. 3), there were no significant differences between the audiovisual and the visual-only or auditory-only stimulus condition (all p > 0.05). Furthermore, post hoc Bonferroni-corrected paired t-tests on the experimental tasks revealed that (i) in each of the audiovisual, visual-only and auditory-only stimulus conditions, the reproducibility ratios for gender/emotion categories were significantly higher for each relevant task (gender categories: the gender or the bi-feature task, left panel of Fig. 3; emotion categories: the emotion or the bi-feature task, right panel of Fig. 3) than for each irrelevant task (gender categories: the number or the emotion task, left panel of Fig. 3; emotion categories: the number or the gender task, right panel of Fig. 3) (all p < 0.05, corrected) and that (ii) in each of the audiovisual, visual-only and auditory-only stimulus conditions, there were no significant differences in the reproducibility ratios for gender/emotion categories between two relevant tasks or between two irrelevant tasks (all p > 0.05).

For each run of the experiment, we further calculated the decoding accuracies of the gender categories (“male” vs. “female”) and the emotion categories (“crying” vs. “laughing”) (see Materials and Methods), which are presented in Fig. S5. The decoding results also reveal the enhancement effect produced by the audiovisual stimuli only for task-relevant features (see Fig. S5).

When the brain is receiving both auditory and visual signals, more reproducible representations may be produced even if no audiovisual integration occurs. We thus conducted a control experiment that included an incongruent audiovisual run for the gender task and one for the emotion task. The experimental procedure for each run was similar to that of the congruent audiovisual run with gender/emotion task of the main experiment except that the audiovisual stimuli were incongruent in the gender or emotion dimension. The experimental results demonstrated that compared with the visual-only and auditory-only stimulus conditions, the incongruent audiovisual stimuli did not enhance the neural representation of the attended features (see the control experiment in the Supplemental Information for details).

### MVPA results for informative voxels, cross-reproducibility ratios, and functional connectivity

By applying an MVPA method to the data collected in the audiovisual condition with bi-feature task, we obtained the informative voxels for gender/emotion category discrimination (see Materials and Methods). The distributions of these informative voxels are presented in Tables 2 and 3 for gender categories and emotion categories, respectively.

**Figure 4:**
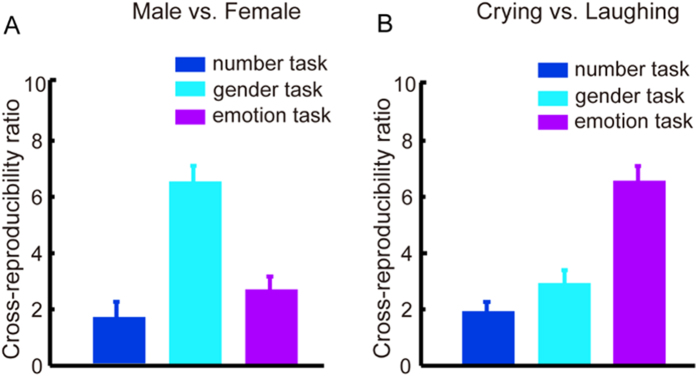
Cross-reproducibility ratios (means and standard errors across all subjects) in the audiovisual stimulus conditions with number, gender and emotion tasks. Left/Right: gender/emotion categories.

Based on the set of the informative voxels for gender/emotion categories, we calculated reproducibility ratios for each of the audiovisual runs with the number, gender and emotion tasks. The average cross-reproducibility ratios across all subjects are presented in Fig. 4. For cross-reproducibility ratios corresponding to gender/emotion categories, one-way ANOVA revealed significant main effects of tasks (number, gender and emotion tasks) (p < 10^−9^, F(2, 8) = 36.97 for gender categories; p < 10^−11^, F(2, 8) = 46.13 for emotion categories). Furthermore, post hoc Bonferroni-corrected paired t-tests demonstrated that the cross-reproducibility ratios were significantly higher for the relevant task than for the irrelevant tasks (gender categories: p < 0.001 corrected, t(8) = 16.23 for gender task vs. number task; p < 0.001 corrected, t(8) = 15.49 for gender task vs. emotion task; emotion categories: p < 0.001 corrected, t(8) = 16.05 for emotion task vs. number task; p < 0.001 corrected, t(8) = 14.36 for emotion task vs. gender task) and that there was no significant difference between the number task and the irrelevant emotion/gender task (all p > 0.05) (Fig. 4). Based on the set of informative voxels for the gender/emotion categories, we also performed gender category and emotion category decoding for each of the audiovisual runs with number, gender and emotion tasks; the corresponding cross-decoding accuracy rates are presented in Fig. S6. From Tables 2 and 3 and Figs 3 and S6, we can conclude the following: (i) the informative voxels in Table 2/Table 3 are involved in the processing of the gender/emotion feature in the audiovisual conditions; (ii) the corresponding voxels in Table 2/Table 3 are informative only when the gender/emotion feature is attended.

**Figure 5:**
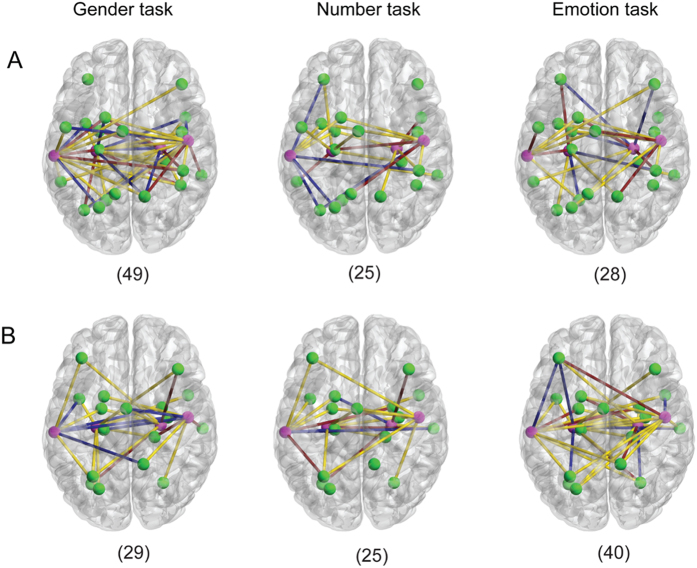
The functional connectivity between the heteromodal areas and the brain areas encoding the gender feature (A) or the emotion feature (B). Green spheres: brain areas from Table 2 in (**A**) or Table 3 in (**B**). Magenta spheres: heteromodal areas. Yellow lines: connections from the heteromodal areas to the informative brain areas. Blue lines: connections from the informative brain areas to the heteromodal areas. Purple lines: connections with bi-direction. Numbers in brackets: total numbers of functional connections.

For the purpose of functional connectivity calculation, we selected four voxel clusters each with size 62 from the heteromodal areas left STS/MTG (cluster center: (−52 −22 8)), right STS/MTG (cluster center: (54 −18 9)), left perirhinal cortex (cluster center: (−26, −20, −22)), and right perirhinal cortex (cluster center: (26, −18, −22)), as described in the related references^10^,^32^. For each of the audiovisual runs with number, gender and emotion tasks, we calculated the functional connectivity with two directions between the heteromodal areas and the informative brain areas in Table 2 (for gender categories) or Table 3 (for emotion categories) via Granger causality analysis at the group level (see Materials and Methods). As shown in Fig. 5, there were more functional connections from the heteromodal areas to the brain areas encoding the gender/emotion feature (Table 2/Table 3) for the relevant task (gender/emotion task) than for the irrelevant tasks (number and emotion/gender tasks). We thus observed that in the audiovisual condition, feature-selective attention enhanced the functional connectivity and thus regulated the information flows from the heteromodal areas to the brain areas encoding the attended feature. Furthermore, this enhancement of the functional connectivity may imply that both the heteromodal areas and the brain areas encoding the attended feature are involved in audiovisual semantic integration.

### Discussion

In the present study, we explored the neural modulation of audiovisual semantic integration by feature-selective attention. During the fMRI experiment, the subjects were instructed to neglect all features, attend to a single feature (gender or emotion), or simultaneously attend to two features (both gender and emotion) of a series of facial movie clips in the visual-only, auditory-only and audiovisual stimulus conditions. To assess the semantic information of a feature encoded in the brain, we calculated a reproducibility ratio for each feature, experimental task and stimulus condition by applying an MVPA method to the fMRI data, and we further analyzed the functional connectivity between the brain areas encoding the semantic feature and the heteromodal areas. Our results suggested that in the audiovisual condition, feature-selective attention may function as a prerequisite for the audiovisual semantic integration of a feature and that the human brain might selectively integrate the semantic information of the attended feature by enhancing the functional connectivity and thus influencing the information flows from the heteromodal areas to the brain areas encoding the feature. Furthermore, the reproducibility ratio may serve as an index for evaluating the audiovisual semantic integration of a feature.

### Feature-selective attention: enhancing the neural representations of the attended features in the audiovisual condition

Considering the audiovisual conditions with number, gender, emotion, and bi-feature tasks, we observed that the reproducibility ratios and decoding accuracy rates were higher for the attended features than for unattended features (Figs 3 and 4, S4–S6). This result indicates that feature-selective attention enhanced the neural representations of the attended features and thus increased both the similarity of the neural activity patterns within a class (e.g., male or female class) and the difference between the two classes of the neural activity patterns (e.g., male vs. female). To focus on relevant information and ignore what is irrelevant, the human brain is equipped with a selection mechanism accomplished by the cognitive function of attention^34^. Specifically, in the visual-only or auditory-only condition, the brain selectively processes one or several features via feature-selective attention^7^,^8^,^9^,^15^,^16^,^17^. Our results revealed that in the audiovisual condition, the feature-selective attention mechanism still permits selective processing of the attended features. In contrast to the visual-only or auditory-only condition, feature-selective attention in the audiovisual condition selectively enhanced the functional connectivity from the heteromodal areas and the brain areas encoding the attended feature (Fig. 5). This enhancement modulated the corresponding information flows and played an important role in achieving the enhancement of neural representations of the attended features in the audiovisual condition.

### Feature-selective attention: a prerequisite for the audiovisual integration of a semantic feature

First, our data analysis results for the experimental runs with the number task supported the conclusion that feature-selective attention is a prerequisite for the audiovisual integration of a semantic feature. As shown in Fig. 2 (A,E), when none of the features of the audiovisual stimuli were attended, audiovisual sensory integration was not observed, not to mention higher level audiovisual semantic integration. Second, using the data for the audiovisual run with the bi-feature task, we separately localized the brain areas associated with the gender and emotion category differentiations (Tables 2 and 3, respectively). Previous studies have demonstrated that some of the selected brain areas, specifically the STS and the fusiform gyrus, are involved in facial information processing^35^,^36^,^37^,^38^. For each of the audiovisual runs with the number, gender and emotion tasks, we calculated cross-reproducibility ratios and cross-decoding accuracy rates for the gender and emotion features using the selected voxels in Tables 2 and 3. We thus demonstrated that these voxels encoded the semantic information of a feature (gender or emotion) only when the feature was attended (Figs 4 and S6). A distributed network including the dorsal medial superior temporal and ventral intraparietal areas is involved in the multisensory integration of visual and vestibular information^39^. Accordingly, we infer that the audiovisual semantic integration corresponding to a feature might be accomplished by a distributed network including the heteromodal areas and the brain areas encoding the feature (Fig. 5). When a feature of an audiovisual object is not attended, our results indicate that the corresponding informative brain areas are not involved in the processing of this feature (Figs 4 and S6), potentially inhibiting the audiovisual semantic integration for this unattended feature.

### Feature-selective audiovisual semantic integration

In this study, from the perspectives of neural information encoding and functional connectivity, we demonstrated the modulation effects of feature-selective attention on audiovisual semantic integration. Specifically, when one or two features of the audiovisual objects were attended, the enhancement of the neural response level in the heteromodal areas of the pSTS/MTG indicated the occurrence of audiovisual sensory integration (Fig. 2B–D,F–H), providing the basis for the audiovisual semantic integration corresponding to the attended features. MVPA analysis demonstrated that for only the attended features, the semantic information encoded in the brain was improved by the audiovisual stimuli compared with the visual-only and the auditory-only stimuli (Figs 3, S4, and S5). We previously considered the case in which a single feature of the stimuli was attended^22^, as in the experiment with the gender and emotion tasks in this study. Compared with the visual-only and auditory-only stimulus conditions, we observed that the congruent audiovisual stimuli enhanced the neural representation of the attended features. However, how this enhancement is implemented in the brain remains unclear. In this study, we extended this conclusion for the cases in which none of the features was attended or more than one feature of the stimulus was attended. Furthermore, the Granger causal connectivity analysis indicated that not only the heteromodal areas but also the brain areas encoding the attended features may be involved in the audiovisual semantic integration. In the audiovisual condition, feature-selective attention enhanced/reduced the functional connectivity from the heteromodal areas and the brain areas encoding the attended/unattended feature (Fig. 5) and therefore modulated the information flows among these areas. This modulation may be responsible for the enhancement of the semantic information of the attended features by the audiovisual stimuli. Through this modulation of feature-selective attention, the human brain may selectively integrate the semantic information for the attended features of the audiovisual facial stimuli. By contrast, for the unattended features, the corresponding audiovisual semantic integration was inhibited.

### Reproducibility ratio: an index for the audiovisual semantic integration of a feature

To form high-level conceptual representations of the semantic features of an audiovisual object, the brain performs audiovisual semantic integration, which may be based on audiovisual integration at the sensory level^10^. Numerous neuroimaging and electrophysiological studies have demonstrated that congruent audiovisual stimuli can enhance neural activities, e.g., in the bilateral superior temporal gyrus (STG)^18^,^19^,^20^,^21^. Conversely, in the audiovisual condition, the enhancement of brain activities in heteromodal areas such as the pSTS/MTG may serve as an indicator of audiovisual sensory integration^4^,^24^,^25^,^26^. Regarding audiovisual semantic integration, numerous studies have discussed the influences of semantic factors on audiovisual integration (see reference^40^ and the references therein). However, no studies have addressed the differentiation of the effects of audiovisual semantic integration for different semantic features. The difficulty may lie in the assessment of the integrated and unintegrated information contained in the brain signals. In this study, we observed that the audiovisual semantic integration effects associated with different feature-selective attention tasks could not be differentiated based on the levels of neural activities in the pSTS/MTG (see Results and Fig. 2). This result is consistent with the function of the pSTS/MTG as a presemantic, heteromodal region for crossmodal perceptual features^10^. MVPA approaches open the possibility of separating and localizing spatially distributed patterns, which generally are too weak to be detected by univariate methods such as GLM^23^,^41^,^42^,^43^. Using an MVPA method, we calculated a reproducibility ratio corresponding to a feature to assess the semantic information encoded in the brain; the corresponding semantic information was enhanced only for the attended features when the audiovisual stimulus condition was compared with the visual-only and auditory-only stimulus conditions (Figs 3, S4 and S5). We thus observed the differential effects of audiovisual semantic integration for the attended and unattended features. Furthermore, the reproducibility ratio might be used as an index for evaluating the audiovisual semantic integration of a feature.

Finally, we describe several limitations of this study to illustrate future directions. First, we employed a relatively complex experimental design, which led to the collection of large amounts of data. For each subject, the collection of the functional and structural MRI data lasted about six hours, not including preparation time. Because of the difficulty in data collection, we used a relatively small number of subjects. But statistically significant experimental results were still obtained. Second, only visual-only, auditory-only and audiovisual facial stimuli were considered in this study. In the future, we must simplify our experimental design, increase the number of subjects, and further consider non-facial stimuli to extend our conclusions.

## Additional Information

**How to cite this article**: Li, Y. *et al.* Selective Audiovisual Semantic Integration Enabled by Feature-Selective Attention. *Sci. Rep.* **6**, 18914; doi: 10.1038/srep18914 (2016).

## Supplementary Material

Supplementary Information

## Figures and Tables

**Table 1:** Tasks associated with selective attention, experimental instructions, and the numbers of the attended features of the visual-only, auditory-only, or audiovisual facial stimuli for each task in the experiment.

Task type	Task description and instruction	Number of attended features
Number task	Attend numbers (i.e., find and count the repeated numbers) while ignoring the visual-only, auditory-only, or audiovisual facial stimuli. Instructions: “Press right-hand key 1 if there are odd numbers of repeats of the numbers; otherwise, press right-hand key 2”.	0
Gender task	Attend the gender feature while ignoring the emotion feature of the stimuli. Instructions: “male: press right-hand key 1; female: press right-hand key 2.”	1
Emotion task	Attend the emotion feature while ignoring the gender feature of the stimuli. Instructions: “crying: press right-hand key 1; laughing: press right-hand key 2”.	1
Bi-feature task	Attend both the gender and emotion features. Instructions: “cry: press left-hand key 1; laugh: press left-hand key 2; male: press right-hand key 1, female: press right-hand key 2”.	2

Note: for the first three tasks, the two right-hand keys were pseudo-randomly assigned to the two categories in each block of a run; for the fourth task, the two left-hand and the two right-hand keys were pseudo-randomly assigned to the gender and the emotion features, whereas the two keys on the left/right-hand side were pseudo-randomly assigned to the two categories in the corresponding feature dimension in each block.

**Table 2:** Distribution of informative voxels for the gender category discrimination (p < 0.05, corrected).

Brain region	Tal coordinates			max weight	Numbers of voxels in the clusters
	x	y	z		
Left Precentral Gyrus	−51	−1	30	0.092	42
Left Fusiform Gyrus	−36	−68	−12	0.084	22
Right Fusiform Gyrus	42	−48	−16	0.066	26
Left Amygdala	−24	−4	−20	0.088	23
Left Parahippocampal Gyrus	−24	−19	−20	0.115	33
Right Hippocampal Gyrus	30	−29	−5	0.054	28
Left Precuneus	−11	−55	42	0.081	43
Right Precuneus	13	−57	50	0.096	52
Left Lingual Gyrus	−18	−60	−5	0.062	24
Left Putamen	−19	7	−2	0.126	32
Right Insula	43	−5	1	0.087	28
Left Anterior Cingulate	−5	−4	42	0.079	25
Left Superior Parietal Lobule	−24	−66	46	0.068	22
Right Inferior Parietal Lobule	44	−36	27	0.074	26
Right Middle Frontal Gyrus	43	34	26	0.083	29
Left Medial Frontal Gyrus	−33	38	−12	0.075	27
Left Superior Temporal Gyrus	−35	2	−19	0.125	32
Right Superior Temporal Gyrus	46	7	−15	0.092	36
Left Middle Temporal Gyrus	−55	−45	−3	0.095	24
Right Middle Temporal Gyrus	59	−40	−15	0.086	29

**Table 3:** Distribution of informative voxels for the emotion category discrimination (p < 0.05, corrected).

Brain region	Tal coordinates			max weight	Numbers of voxels in the clusters
	x	y	z		
Right Precuneus	12	−50	52	0.087	23
Left Middle Frontal Gyrus	−38	36	30	0.067	26
Right Middle Frontal Gyrus	40	27	43	0.084	32
Right Middle Temporal Gyrus	60	−21	−10	0.092	35
Left Superior Temporal Gyrus	−40	3	−21	0.125	28
Right Superior Temporal Gyrus	48	6	−18	0.083	42
Left Fusiform Gyrus	−30	−66	−10	0.091	27
Right Fusiform Gyrus	28	−65	−7	0.0825	36
Left Parahippocampal Gyrus	−20	−22	−12	0.096	28
Right Parahippocampal Gyrus	21	−28	−9	0.093	32
Right Hippocampal Gyrus	32	−12	−17	0.087	27
Left Amygdala	−22	−5	−16	0.072	30
Right Amygdala	27	−4	−19	0.067	26
Left Putamen	−20	5	−3	0.0813	32
Left Anterior Cingulate	−2	−5	38	0.065	29
Left Superior Parietal Lobule	−24	−70	44	0.084	36
Right Superior Parietal Lobule	29	−60	49	0.097	25
