# Supplementary material for: Selective Audiovisual Semantic Integration Enabled by Feature-Selective Attention
Source: Sci Rep. 2016 Jan 13;6:18914. doi: 10.1038/srep18914 (PMC4725371; doi:10.1038/srep18914)
Supplement: Supplementary Information [file srep18914-s1.doc]

**Selective Audiovisual Semantic Integration Enabled by**

**Feature-Selective Attention**

Yuanqing Lia (corresponding author), Jinyi Longa, Biao Huangb, Tianyou Yua, Wei Wua, Peijun Lib, Fang Fangc, Pei Sund

a. Center for Brain Computer Interfaces and Brain Information Processing, South China University of Technology, Guangzhou, 510640, China (e-mail: [auyqli@scut.edu.cn](mailto:auyqli@scut.edu.cn); phone: 86-20-87114390)

b. Department of Radiology, Guangdong General Hospital, Guangzhou, 510080, China.

c. Department of Psychology and Key Laboratory of Machine Perception (Ministry of Education), Peking University, Beijing 100871, China.

d. Department of Psychology, School of Social Sciences, Tsinghua University, Beijing, 100084, China.

**Contact:** Yuanqing Li, [auyqli@scut.edu.cn](mailto:auyqli@scut.edu.cn)

**Supplemental Information：**

**Multi-variate pattern analysis (MVPA).** The following Figures S1 and S2 illustrate the MVPA procedures for the calculation of the reproducibility ratios, decoding accuracy rates, cross-reproducibility ratios, and functional connectivity.

**
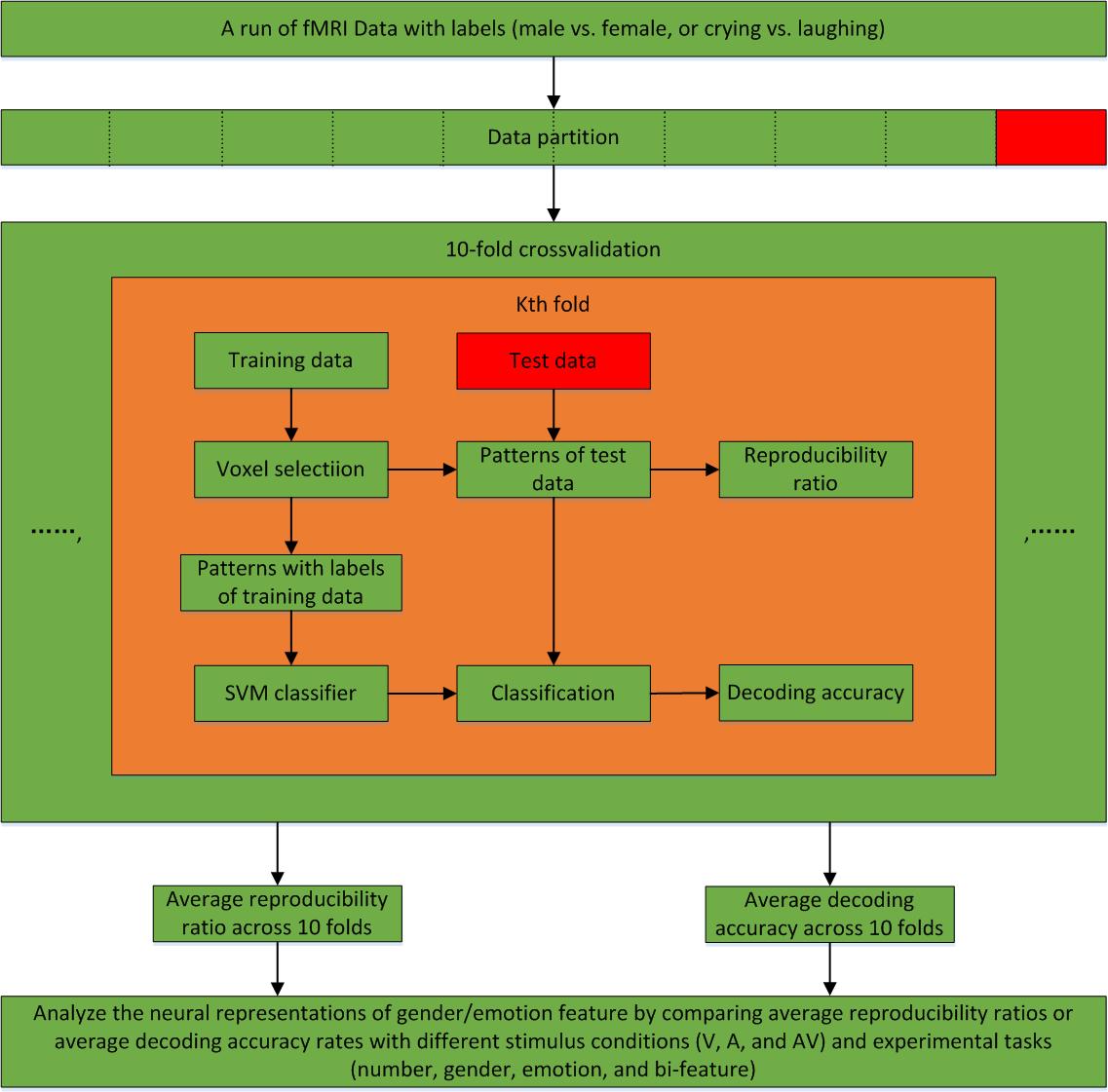
**

Figure S1: MVPA procedure for the calculation of the reproducibility ratio and decoding accuracy in an experimental run.

**
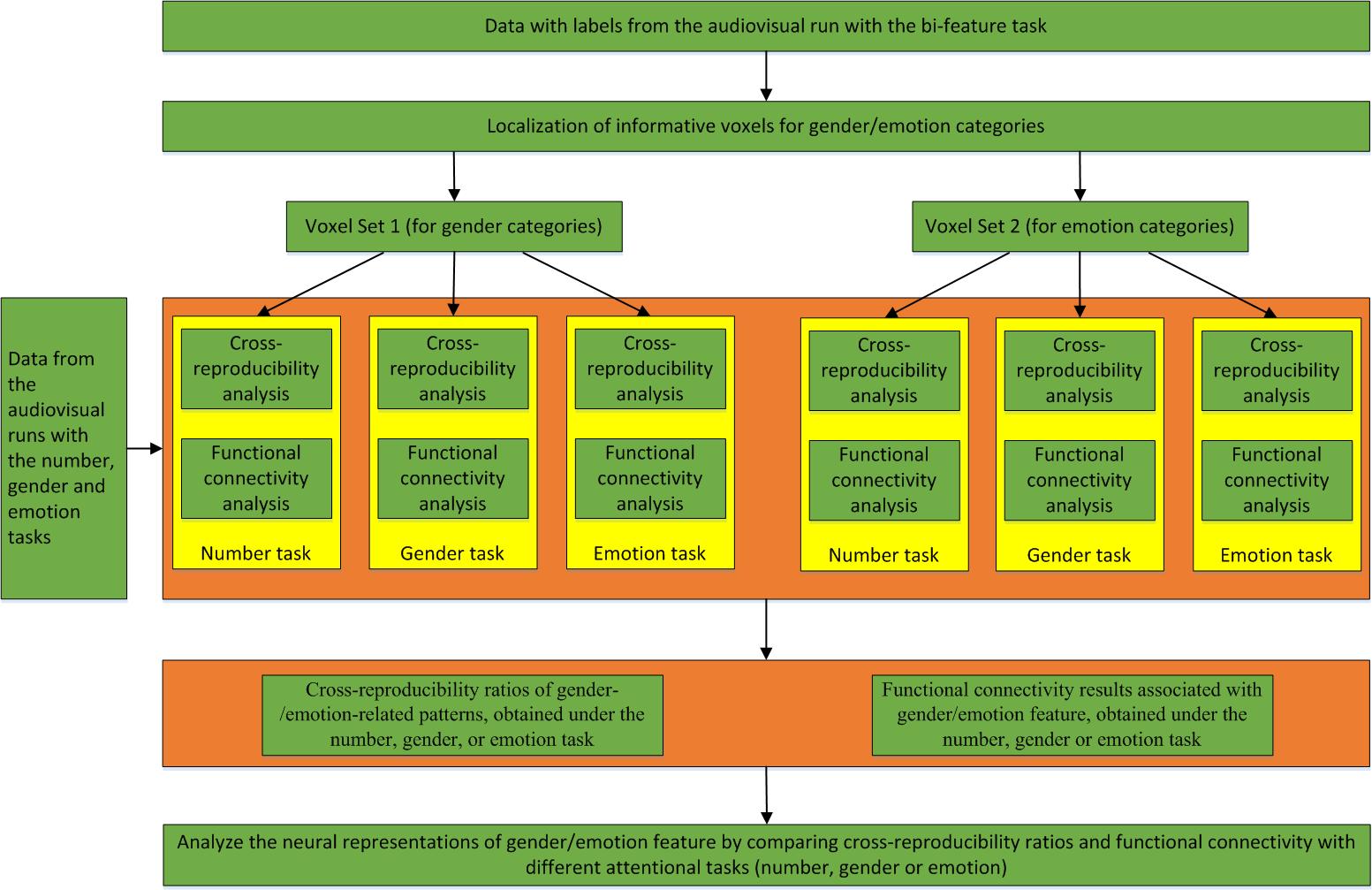
**

Figure S2: MVPA procedure for the calculation of the cross-reproducibility ratio and functional connectivity.

**Behavioral results.** Figure S3 shows the behavioral results, i.e., the reaction times (RTs) and the percentages of correct judgment from the fMRI experiment. Note that in an experimental trial, if the subjects did not press the keys in the last 6 s, this trial was counted as a failure in our behavior data analysis. For the runs with number task (Figure S3A and S3E), one-way repeated measures ANOVA revealed that there were no significant main effects of stimulus condition (the audiovisual, visual-only and auditory-only conditions) on both reaction time (RT) (p=0.93, F(2, 8)=0.07) and the percentage of correct judgment (p=0.53, F(2, 8) =0.65). This is reasonable since the number judgment task in this experiment was not related to the stimulus conditions. Additionally, the behavior accuracy rates were <50% , as shown in Figure S3(E). The reason was that there existed several trials of each number run, in which the subjects hesitated with their judgments and failed/discarded to press the keys in the last 6 s.

For the runs with gender task, one-way repeated measures ANOVA revealed that there was a significant main effect of stimulus condition (the audiovisual, visual-only and auditory-only conditions) on RT (p<0.0001, F(2, 8)=21.202) (Figure S3B). Post-hoc Bonferroni-corrected paired t-tests showed that the RT was significantly lower for the audiovisual stimulus condition than for the auditory-only stimulus condition (p<0.001 corrected). There was no significant difference between the RT for the audiovisual stimulus condition and that for the visual-only stimulus condition (p>0.05). One-way repeated measures ANOVA also showed a significant main effect of stimulus condition on the percentage of correct judgment (p<0.0001, F(2, 8)=16.922) (Figure S3F). Post-hoc Bonferroni-corrected paired t-tests showed that the percentage of correct judgment was significantly higher for the audiovisual stimulus condition than for the auditory-only stimulus condition (p<0.001 corrected). There was no significant difference between the percentage of correct judgment for the audiovisual stimulus condition and that for the visual-only stimulus condition (p>0.05).

For the runs with emotion task, one-way repeated measures ANOVA revealed that there was a significant main effect of stimulus condition (the audiovisual, visual-only and auditory-only conditions) on RT (p<0.0001, F(2, 8)=12.500) (Figure S3C). Post-hoc Bonferroni-corrected paired t-tests showed that the RT was significantly lower for the audiovisual stimulus condition than for the auditory-only stimulus condition (p<0.001 corrected). There was no significant difference between the RT for the audiovisual stimulus condition and that for the visual-only stimulus condition (p>0.05). One-way repeated measures ANOVA also showed a significant main effect of stimulus condition on the percentage of correct judgment (p<0.0001, F(2, 8) = 17.852) (Figure S3G). Post-hoc Bonferroni-corrected paired t-tests showed that the percentage of correct judgment was significantly higher for the audiovisual stimulus condition than for the visual-only stimulus condition (p < 0.04 corrected) and for the auditory-only stimulus condition (p< 0.001 corrected).

For the runs with bi-feature task, one-way repeated measures ANOVA revealed that there was no a significant main effect of stimulus condition (the audiovisual, visual-only and auditory-only conditions) on RT (p=0.36, F(2, 8)=1.07), although the average RT was lower for the audiovisual stimulus condition than for the visual-only or auditory-only stimulus condition, as shown in Figure S3D. One-way repeated measures ANOVA showed that there was a significant main effect of stimulus condition on the percentage of correct judgment (p<0.01, F(2, 8) =6.06) (Figure S3H). Post-hoc Bonferroni-corrected paired t-tests showed that the percentage of correct judgment was significantly higher for the audiovisual stimulus condition than for the auditory-only stimulus condition (p<0.01 corrected). There was no significant difference between the percentage of correct judgment for the audiovisual stimulus condition and that for the visual-only stimulus condition (p>0.05). Additionally, the bi-feature task led to slower responses compared with the gender and emotion tasks. This was because in a trial with the bi-feature task, the subjects made the judgments of both gender and emotion features and pressed two response keys (one is a left-hand key and the other is a right-had key), whereas they made the judgment of only one feature and pressed only one right-hand key in a trial with the gender or emotion task.


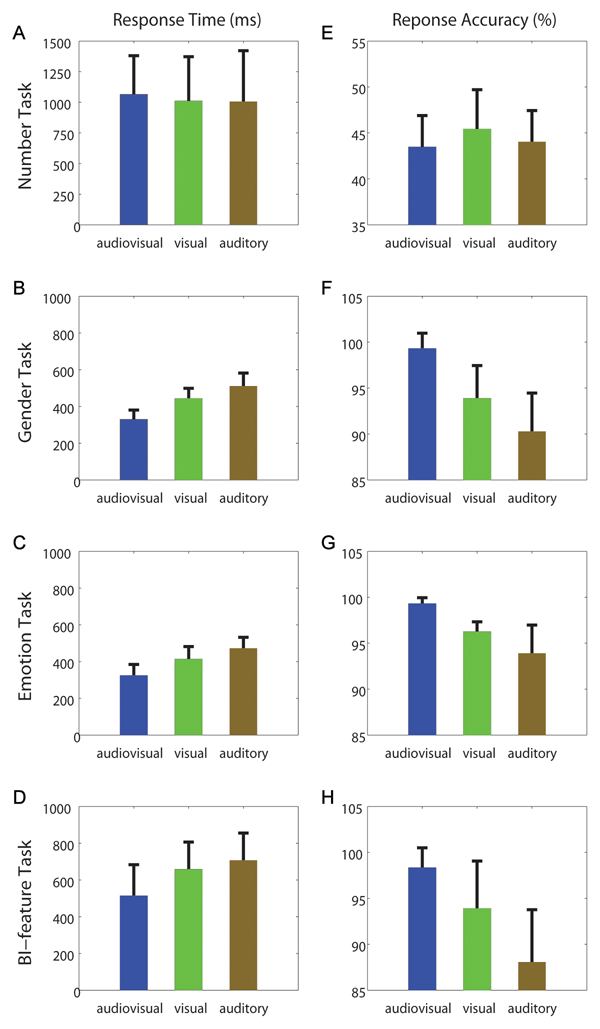


Figure S3. Behavioral results for the fMRI experiment: A and E): runs with the number task; B and F): runs with the gender task; C and G): runs with the emotion task; D and H): runs with the bi-feature task. Left: Reaction times (mean and standard error) in the audiovisual, visual-only, and auditory-only stimulus conditions. Right: Percentages of correct judgment (mean and standard error) in the audiovisual, visual-only, and auditory-only stimulus conditions.

**Reproducibility ratio curves.** We systematically varied the number of selected voxels from 25 to 1500 for calculating the reproducibility ratios. The obtained reproducibility ratio curves are shown in Figure S4.


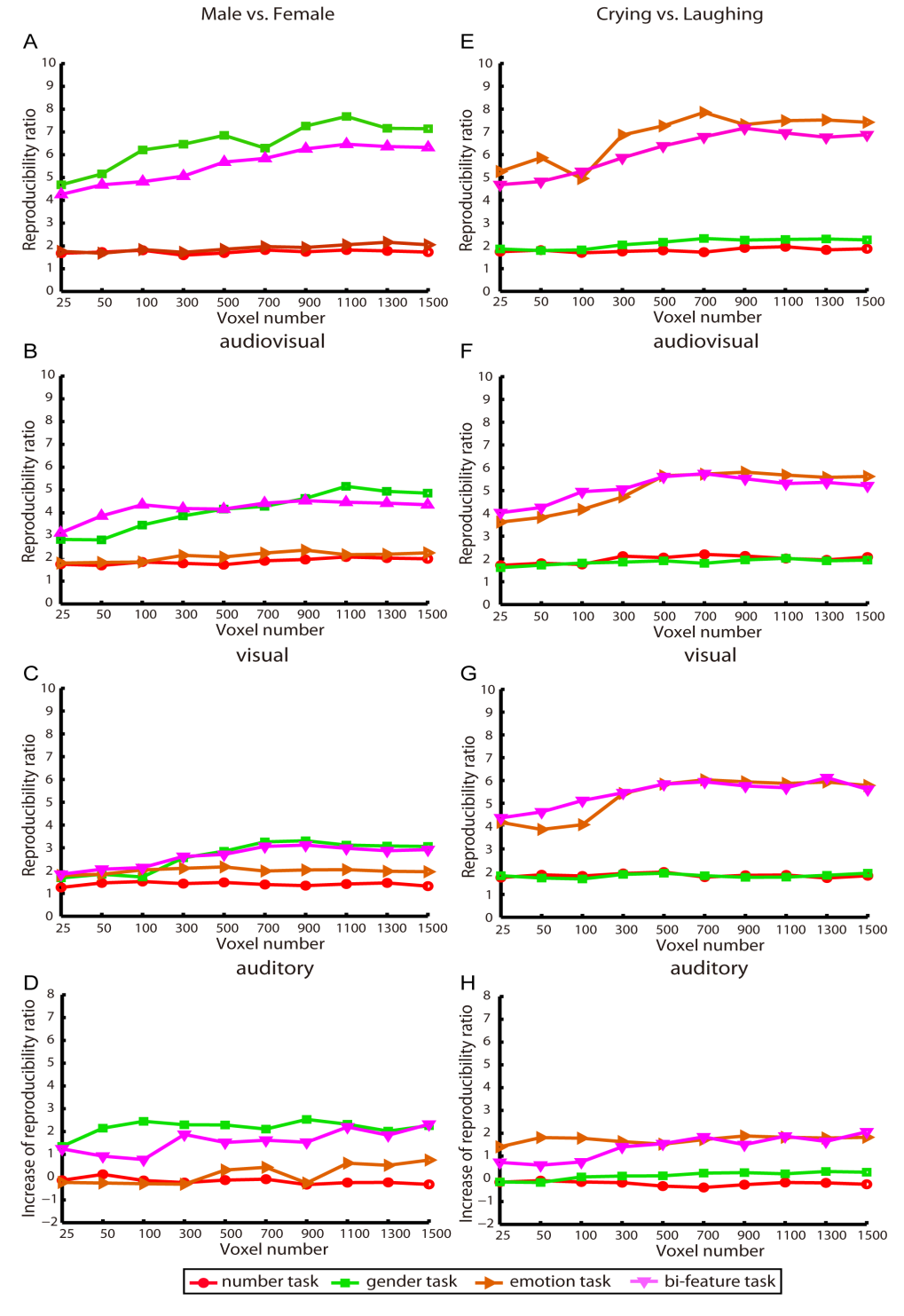


Figure S4. Average reproducibility ratio curves across all subjects with respect to the number of selected voxels and the corresponding comparison results (see Methods). Left/Right: gender/emotion categories. The first three rows: audiovisual, visual-only and auditory-only stimulus conditions, respectively; the fourth row: curves showing the differences between the average reproducibility ratios in the audiovisual stimulus condition and the maximum of the average reproducibility ratios in the visual-only and auditory-only stimulus conditions for each experimental task and for the gender/emotion categories.

**Decoding results.** For each experimental run, we separately decoded the gender categories (“male” and “female”) and the emotion categories (“crying” and “laughing”) of the stimuli from the collected fMRI signals using the MVPA method (see Experimental Procedures). We systematically varied the number of the selected voxels from 25 to 1500 for decoding the gender/emotion categories and the obtained results are shown in Figure S5. We found that when the subjects attended to the gender, emotion or both features, the decoding accuracy of the task-relevant feature (gender or emotion) was higher for the audiovisual stimulus condition than for the visual-only or the auditory-only stimulus condition. This enhancement effect produced by the audiovisual stimuli was not observed for task-irrelevant features. In the following, as an example, we used 1500 selected voxels to present the statistical test results.


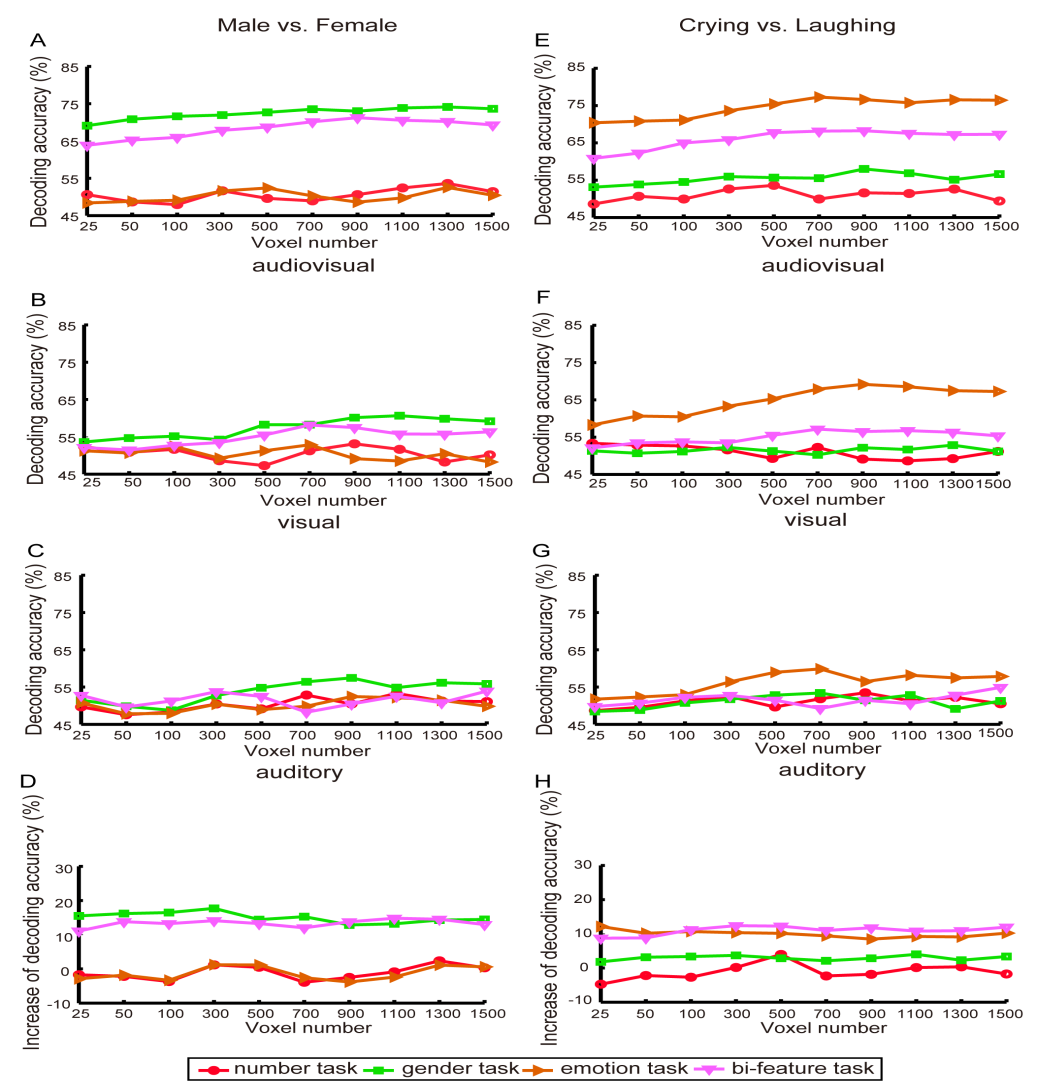


Figure S5. Average decoding accuracy curves across all subjects with respect to the number of selected voxels and the corresponding comparison results. Left/Right: decoding gender/emotion categories. The first three rows: the audiovisual, visual-only and auditory-only stimulus conditions, respectively. The fourth row: curves showing the differences between the average decoding accuracies in the audiovisual stimulus condition and the maximum of the average decoding accuracies in the visual-only and auditory-only stimulus conditions for each experimental task and for the gender/emotion categories.

Regarding the gender/emotion decoding accuracies, a two-way repeated measures ANOVA revealed that there were a significant main effects of stimulus condition (gender category decoding: p<10^-16^, F(2, 8)=56.09; emotion category decoding: p<10^-12^, F(2, 8)=33.59) and experimental task (gender category decoding: p<10^-17^, F(3, 8)=71.55; emotion category decoding: p<10^-17^, F(3, 8)=72.35). There was also a significant interaction effect between the two factors of stimulus condition and experimental task (gender category decoding: p<10^-17^, F(6, 8)=29.02; emotion category decoding: p<10^-17^, F(6, 8)= 31.26). Post-hoc Bonferroni-corrected paired t-tests on the stimulus conditions showed that (i) for the relevant tasks (gender category decoding: the gender and the bi-feature tasks; emotion category decoding: the emotion and the bi-feature tasks), the decoding accuracy rates were significantly higher for the audiovisual stimulus condition than for the visual- or auditory-only stimulus condition (all p<0.001 corrected); and (ii) for the irrelevant tasks (gender category decoding: the number and the emotion tasks; emotion category decoding: the number and the gender tasks), there were no significant differences between the audiovisual and the visual-only or the auditory-only stimulus condition (all p>0.05).

**Cross-decoding accuracies.** Using the data collected in the audioviual run with bi-feature task, we obtained the informative voxels for gender/emotion category discrimination (see Methods). The distribution of these informative voxels were shown in Table 1 (for gender categories) and Table 2 (for emotion categories). Based on the set of the informative voxels for gender categories, we separately performed gender decoding for the audiovisual runs with the number, the gender and the emotion tasks. We also separately performed emotion decoding for the audiovisual runs with the number, the gender and the emotion tasks, based on the set of the informative voxels for emotion categories.

The average cross-decoding results across all the subjects are shown in Figure S6. For the gender and the emotion category cross-decoding, a one-way ANOVA revealed that there were significant main effects of tasks (number, gender and emotion tasks: p<$10^{-10}$, F(1, 8)=55.6 for gender cross-decoding; p<$10^{-9}$, F(1, 8)=42.09 for emotion cross-decoding). Furthermore, Post-hoc Bonferroni-corrected paired t-tests showed that the decoding accuracy rates were significantly higher for the relevant tasks than for the irrelevant tasks **(**gender cross-decoding: p < 0.001 corrected, t(8) =19.62 for gender task vs. number task; p < 0.001 corrected, t(8) = 16.39 for gender task vs. emotion task; emotion cross-decoding: p < 0.001 corrected, t(8) =21.05 for emotion task vs. number task; p < 0.001 corrected, t(8) = 18.36 for emotion task vs. gender task). There was no significant difference between the number task and the irrelevant emotion/gender task for the gender/emotion cross-decoding.


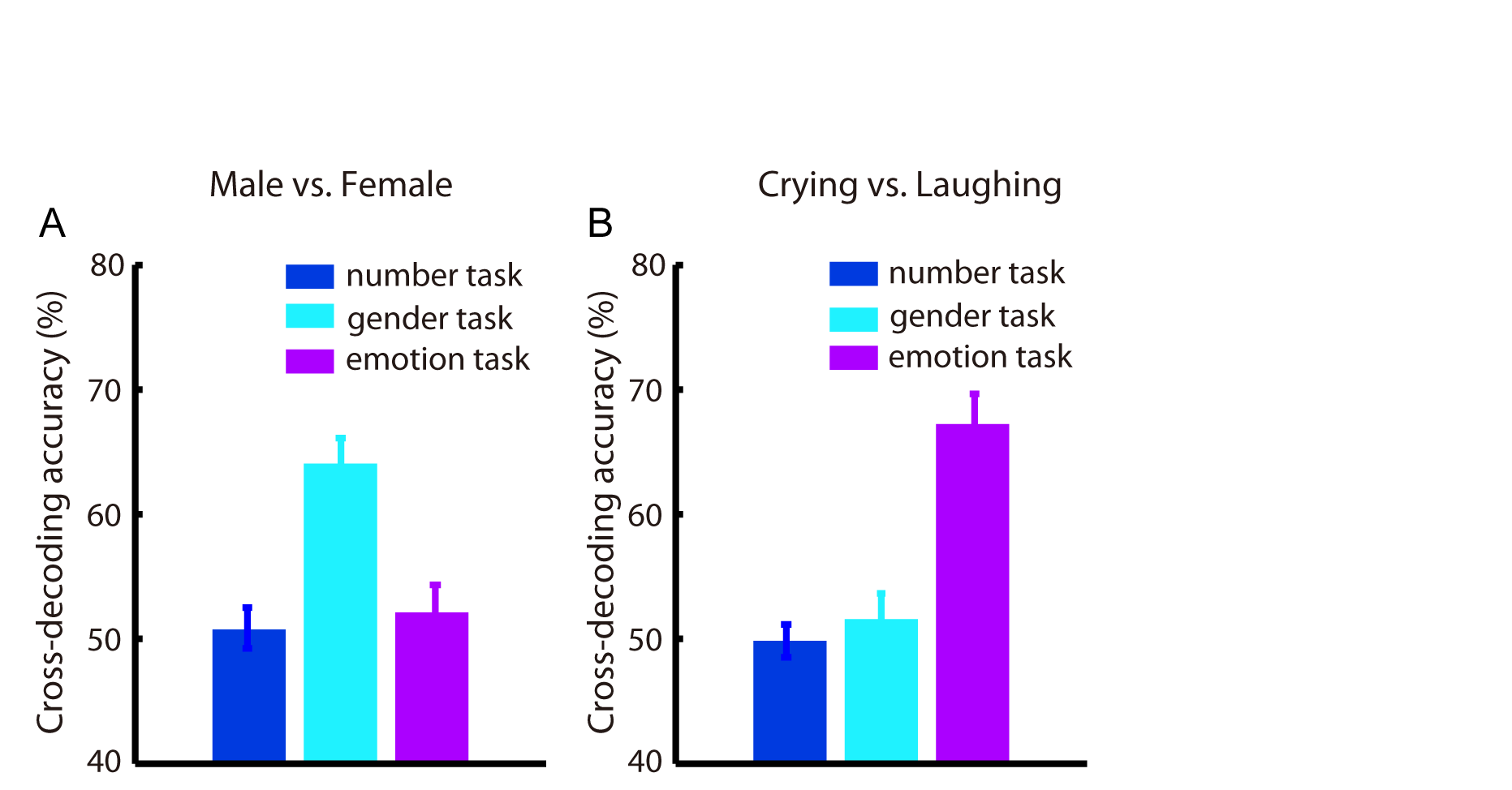


Figure S6: Cross-decoding results (means and standard errors across all subjects) in the audiovisual runs with the number, gender and emotion tasks. Left/Right: decoding gender/emotion categories.

**A control experiment.** After the main experiment, each subject performed a control experiment in a day. The control experiment included two incongruent audiovisual runs for gender and emotion tasks respectively. We constructed 160 incongruent audiovisual stimuli, in which 80 for gender feature and the other for emotion feature. Specifically, an incongruent audiovisual stimulus for gender/emotion feature was obtained by pairing a video with an audio which were from different people, incongruent in gender/emotion feature, but congruent in emotion/gender feature. Note that the 80 incongruent audiovisual stimuli for gender/emotion feature could be semantically partitioned into two groups (gender feature: male face + female voice vs. female face + male voice; emotion feature: crying face + laughing voice vs. laughing face + crying voice). The experimental procedure, data collection, and data analysis for each incongruent audiovisual run were similar to those in the congruent audiovisual run for gender or emotion task of the main experiment in this study (see Materials and Methods). Not that prior to the functional scanning, a 3D anatomical T1-weighted scan (FOV, 280 mm; matrix, 256 × 256; 128 slices; slice thickness: 1.8 mm) was also acquired for each subject.

*GLM analysis.* In order to check whether the audiovisual sensory integration occurred or not in the incongruent audiovisual conditions, we performed the general linear model (GLM) analysis at the group level using the data of the two incongruent audiovisual runs and four unimodal runs (visual-only and auditory-only runs for gender task, and visual-only and auditory-only runs for emotion task). According to the statistical criterion [AV>max (A,V) (p<.05, FWE-corrected)] ∩ [V>0 or A>0 (p<0.05, uncorrected)], no brain areas were identified to show the enhancement of neural responses for the gender or emotion task in the incongruent audiovisual condition. This implied that audiovisual sensory integration did not occur for the two incongruent audiovisual runs.

*Reproducibility results.* We applied the MVPA method (see Materials and Methods) to the data collected in the incongruent audiovisual conditions. Specifically, for the incongruent audiovisual run with gender/emotion task, we calculated an average reproducibility ratio with 1500 selected voxels corresponding to the gender/emotion categories (male face + female voice vs. female face + male voice, or crying face + laughing voice vs. laughing face + crying voice) of the stimuli. We also systematically varied the number of selected voxels from 25 to 1500 for calculating the average reproducibility ratios. Figure S7 shows the reproducibility results for the incongruent audiovisual runs as well as the visual-only and auditory-only runs of the main experiment for the purpose of comparison. Using the reproducibility results obtained with 1500 voxels, we compared the incongruent audiovisual run with the visual-only and auditory-only runs for the gender/emotion task and found no significant difference (gender task: p=0.37, F(2,8)=1.56; emotion task: p>0.54, F(2,8)=1.02, one-way repeated measures ANOVA).


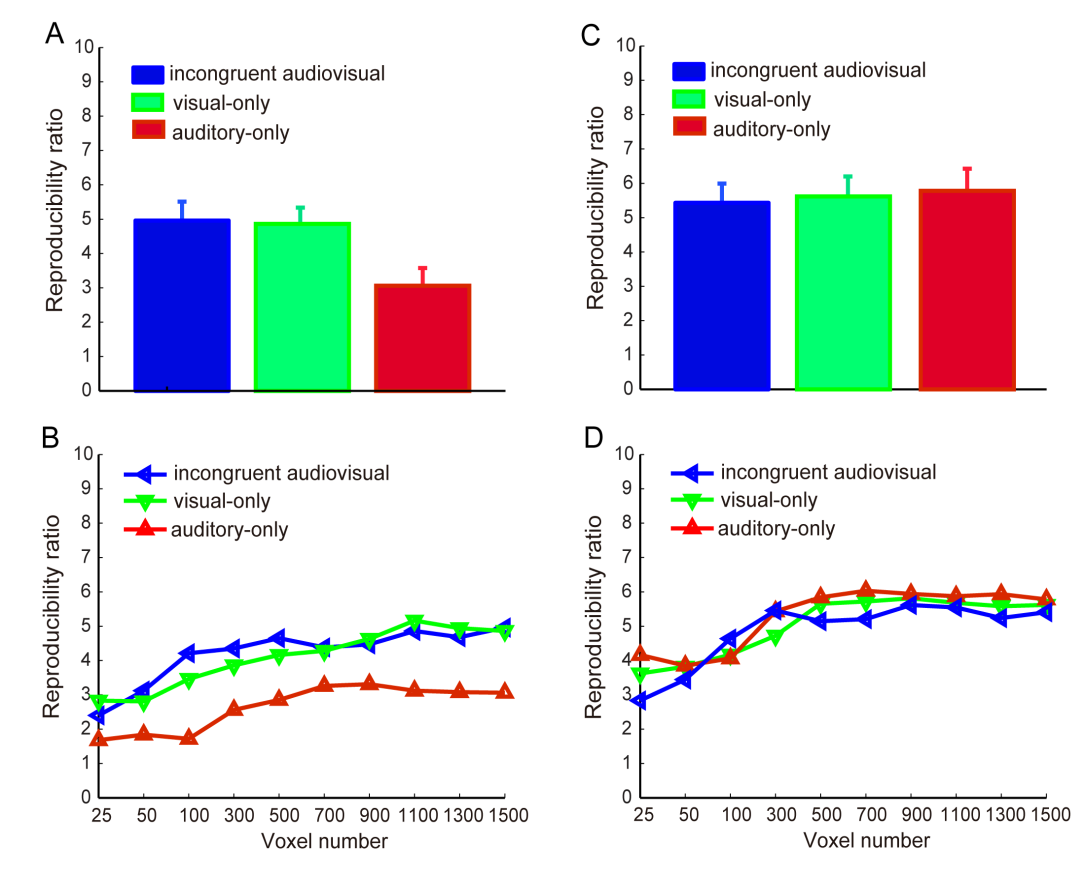


*Decoding results.* Using the MVPA method (see Materials and Methods), we calculated the average decoding accuracy of the gender categories (male face + female voice vs. female face + male voice) for the incongruent audiovisual run with gender task, and the average decoding accuracy of the emotion categories (crying face + laughing voice vs. laughing face + crying voice) for the incongruent audiovisual run with emotion task. Figure S8 shows the decoding results for the incongruent audiovisual runs as well as the visual-only and auditory-only runs of the main experiemnt for the purpose of comparison. Using the decoding accuracies obtained with 1500 voxels, we compared the incongruent audiovisual run with the visual-only and auditory-only runs for the gender/emotion task and found no significant difference (gender decoding: p=0.48, F(2,8)=1.24; emotion decoding: p=0.28, F(2,8)=1.83, one-way repeated measures ANOVA).


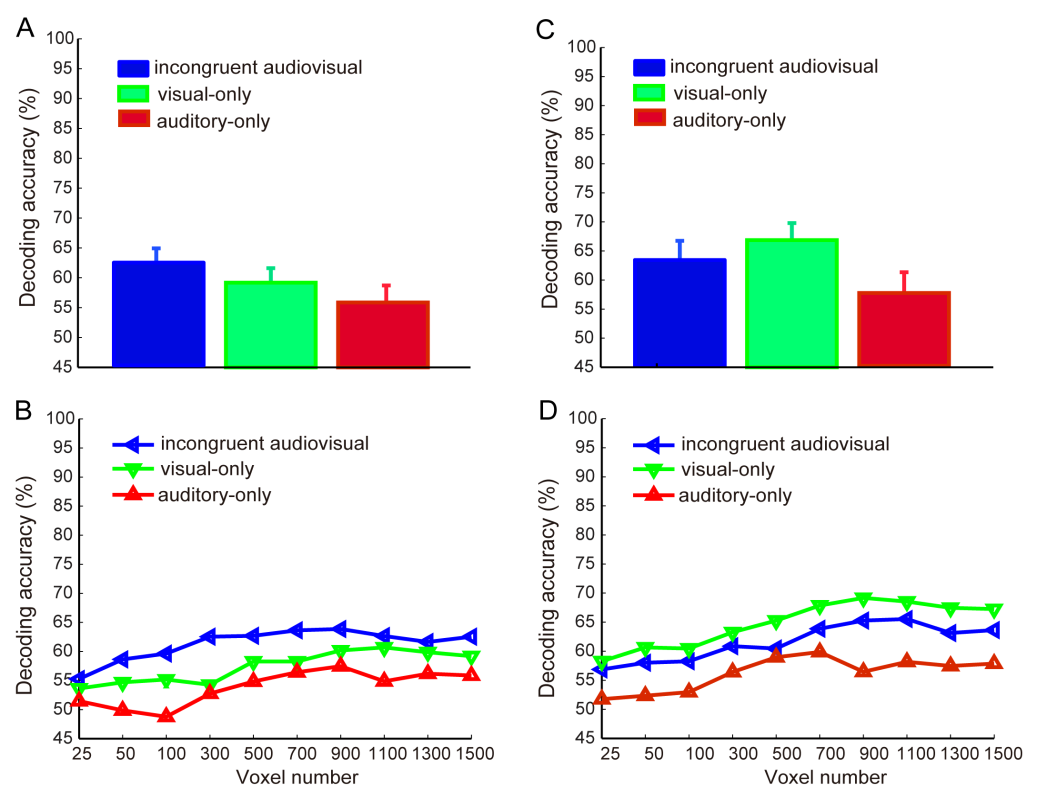


Figure S8. Average decoding accuracy rates across all subjects obtained in the incongruent audiovisual, visual-only, and auditory-only conditions for the gender (A and B) or emotion task (C and D). Left/Right: gender/emotion category decoding. First row: the average decoding accuracy rates calculated using 1500 selected voxels. Second row: the decoding accuracy curves with respect to the number of selected voxels.
